# Detection of clonal mast cell disease in wasp venom allergic patients with normal tryptase

**DOI:** 10.1002/clt2.12174

**Published:** 2022-09-07

**Authors:** Merel C. Onnes, Abdulrazzaq Alheraky, Martijn C. Nawijn, Tim E. Sluijter, André B. Mulder, Suzanne Arends, Hanneke N. G. Oude Elberink

**Affiliations:** ^1^ Department of Internal Medicine Division of Allergology University Medical Center Groningen University of Groningen Groningen The Netherlands; ^2^ Department of Pathology and Medical Biology University Medical Center Groningen University of Groningen Groningen The Netherlands; ^3^ Groningen Research Institute for Asthma and COPD (GRIAC) Research Institute University Medical Center Groningen Groningen The Netherlands; ^4^ Department of Laboratory Medicine University Medical Center Groningen University of Groningen Groningen The Netherlands; ^5^ Department of Rheumatology and Clinical Immunology University Medical Center Groningen University of Groningen Groningen The Netherlands

**Keywords:** insect venom, *KIT* D816V analysis in peripheral blood, mastocytosis, REMA score, tryptase

## Abstract

**Background:**

Clonal mast cell disease (CMD) is an underlying aggravating condition in wasp venom allergy (WVA) which requires a different treatment strategy. CMD is increasingly recognized in patients with normal basal serum tryptase (bsT). However, methods to identify at risk patients have not yet been assessed in large cohorts of WVA patients with normal bsT.

**Methods:**

This retrospective study evaluated the reliability of the REMA score in detecting CMD in a cohort of grade IV WVA patients with normal bsT and assessed the added value of other clinical parameters, *KIT* D816V mutation analysis in peripheral blood (PB) and the diagnosis of hereditary alpha tryptasemia (HAT). All patients had a conclusive bone marrow evaluation that demonstrated or excluded underlying CMD.

**Results:**

In total 35 CMD and 96 non‐CMD patients were included. REMA score had a sensitivity of 72% (95% CI 56%–88%) and specificity of 79% (95% CI 70%–87%) in this cohort. Loss of consciousness during systemic reaction and bsT between 6.3 and 11.4 ng/ml were additional parameters independently associated with CMD. Sensitivity of *KIT* in PB was relatively low, 56% (95% CI 36%–75%), but had added value as screening method in patients with a low REMA score due to 100% specificity.

**Conclusion:**

The REMA score is a relatively reliable method to detect patients at risk of CMD among WVA patients with normal bsT. *KIT* mutation analysis in PB could serve as additional screening method in patients with low REMA scores.

## 1 INTRODUCTION

Hymenoptera venom allergy (HVA) can cause severe, potentially life‐threatening reactions to insect venom. Five years of venom immunotherapy (VIT) provides lasting protection against new episodes of anaphylaxis.^1^ Unfortunately, this does not apply to patients with underlying clonal mast cell disease (CMD). Although most CMD patients are protected during treatment,^2^ the effect wears off after cessation of VIT.^3^ Severe, even fatal reactions have been reported after discontinuation of VIT in CMD patients.^4^, ^5^ Therefore, a lifelong indication for VIT is given to CMD patients,^4^, ^6^ and identification of CMD patients is essential.

Definitive diagnosis of CMD is made through bone marrow evaluation (BME), an invasive procedure that is not always available to allergists. Therefore, there is a need for a sensitive screening method to detect HVA patients at risk of underlying CMD.

In the past, BMEs were mainly conducted in patients with elevated basal serum tryptase (bsT, cut‐off ≥11.4 ng/ml). In recent years, however, the occurrence of CMD among patients with normal bsT has been increasingly recognized.^4^, ^7^ As the majority of HVA patients have normal bsT levels reliable screening methods for this group are highly important. Therefore, we assessed potential risk factors in a bone marrow (BM) evaluated cohort of normal bsT patients. We compared characteristics of CMD and non‐CMD patients and evaluated detection methods previously described in elevated bsT cohorts.

So far, the largest studies on risk factors for CMD in BM evaluated resulted in the REMA score.^8^, ^9^, ^10^ The REMA score uses clinical parameters and bsT levels to detect CMD and is therefore an accessible tool with low costs. It was developed in patients with mast cell activation symptoms in the absence of skin lesions suggesting mastocytosis. Subsequently it was also specifically assessed in HVA patients. In a HVA cohort with 133 patients, of whom only 10 with normal bsT, sensitivity was 92% and specificity 67%.^10^

Aside from the REMA score the *KIT* D816V mutation analysis in peripheral blood (*KIT* in PB) has been proposed as a screening method for CMD, both separately and in combination with the REMA score.^11^, ^12^, ^13^ There are no studies that validated this tool specifically for HVA patients by assessing its sensitivity and specificity in large BM evaluated cohorts. However, two studies included a substantial share of HVA patients. Most promising results are reported by Kristensen et al.^11^ In 32 patients suspected of CMD without maculopapular cutaneous mastocytosis (MPCM), including 18 HVA patients, 22 were diagnosed with CMD, 10 were not. Sensitivity of the analysis was 82%. This high sensitivity could not be confirmed by a Belgian study.^12^ In 74 patients suspected of CMD without MPCM, including 45 with HVA, 30 were diagnosed with (indolent) systemic mastocytosis (SM) or monoclonal mast cell activation syndrome (MMAS). Sensitivity of the analysis was 52%. In spite of these conflicting results on sensitivity, specificity was 100% in both studies.^11^, ^12^ The Belgian study suggests that the combination of REMA score and peripheral *KIT* mutation analysis could be valuable.^12^ A third relevant study routinely performed *KIT* in PB in a large HVA cohort, including 132 patients with severe reactions and normal bsT and describes patient characteristics of *KIT* in PB positive and negative patients.^14^ Unfortunately, however, outcomes of *KIT* in PB were not BM controlled. Given the conflicting evidence on the sensitivity of the *KIT* assay in PB it remains unclear what proportion of CMD patients was missed in this study. Therefore it is unsuitable for adequate analysis of risk factors for CMD.

Lastly, in recent years there has been an increasing interest in the presence of hereditary alpha tryptasemia (HAT) in HVA patients, its correlation with severity of reactions and its potential value in detecting CMD.^14^, ^15^

In our study, the largest BM evaluated cohort, we aim to evaluate which approach is most suitable to detect CMD in patients with severe HVA and normal bsT. Primarily we assessed the REMA score, given its high sensitivity in elevated bsT patients, its accessibility and low costs. Furthermore, through multivariate analysis we assessed whether other clinical parameters could optimize this score for normal bsT patients. Also, we assessed sensitivity and specificity of *KIT* in PB and assessed whether the HAT assay could help to detect CMD within this population. We aim to develop an approach that detects most CMD cases whilst reducing the number of required BMEs.

## 2 METHODS

### 2.1 Study population

This retrospective explorative study assessed all patients who suffered severe systemic reactions to wasp venom (*Vespula vulgaris* and *Vespula germanica*), lacked MPCM and first visited our hospital, a tertiary referral center and national center for expertise on CMD, between 2011 and 2020.

Our analysis was limited to wasp venom allergy (WVA). Bee (*Apis mellifera*) and Polistes dominula venom allergic patients were not assessed due to low frequency of these diagnoses in our center. Patients with MPCM were excluded as this is a clear indication for BME, requiring no further risk assessment. Presence or absence of MPCM was assessed by physicians familiar with mastocytosis.

Systemic reactions (SRs) were graded according to the Müller criteria.^16^ We included patients with grade IV reactions as we do not routinely test for CMD in grade I to III patients in our center. Grade IV reactions include cardiovascular involvement manifested by drop in blood pressure, presyncope, loss of consciousness, cyanosis or incontinence for urine and/or faeces. Reactions that included loss of consciousness were classified as IVb, otherwise they were graded as IVa.

Even though our primary objective concerned patients with normal bsT levels, we included both patients with normal and with elevated bsT. The elevated bsT cohort was used to compare reliability of the REMA score in our population to the previously, elevated bsT dominated, studies.

Patients were excluded if they lacked a conclusive BME. To assess sampling bias and generalizability of the results, we compared patient characteristics of in‐ and excluded patients with normal bsT.

Patient inclusion and informed consent procedure was conducted according to Dutch legislation and ethical regulations.

### 2.2 Evaluation for CMD

Clonal mast cell disease can be divided into (indolent) SM (ISM) and MMAS. SM is diagnosed according to the WHO criteria.^17^ MMAS patients do not fulfill all SM criteria, but show mast cell clonality through a *KIT* mutation and/or aberrant expression of CD25 and/or CD2.^18^ Collection and evaluation of bone marrow biopsy and aspirate were conducted according to previously described methods.^19^

Since 2016 we routinely assessed patients with normal bsT for CMD, as studies had then showed that also among grade IV patients with normal bsT a large proportion suffered from underlying CMD.^4^, ^7^ In addition, from that time onward BME was also offered to patients who returned for check‐up after 5 years of VIT. Patients with elevated bsT were routinely evaluated for CMD at their first visit during the entire inclusion period (2011–2020). Due to the retrospective nature of the study disease status was known to the assessor of outcome variables.

### 2.3 REMA score

The REMA score was applied as described by Álvarez‐Twose et al., using a cut‐off of ≥2 as a high risk score for CMD.^8^ The REMA score incorporates gender (+1 for males, −1 for females), skin symptoms during the SR (+1 if absent, −2 if present), (pre)syncope during the SR (+3 if present) and bsT levels (−1 for levels <15 ng/ml, +2 for levels >25 ng/ml) for CMD risk classification. For normal bsT (−1) patients with grade IV (+3) reactions this means that the presence or absence of skin features is decisive for the REMA score category.

Skin symptoms during the SR include pruritus, urticaria and/or angioedema. Data were retrieved from emergency room reports, referral letters or from history taking. If a patient suffered from at least one of these symptoms, skin features were scored as present.

### 2.4 *KIT* D816V mutation analysis in peripheral blood

The presence of the *KIT* D816V mutation in genomic DNA of PB leucocytes was examined in a subgroup of patients by using a quantitative real‐time (qPCR) assay as described by Kristensen et al.^20^ with a LightCycler 480 II real‐time PCR System (Roche Diagnostics Deutschland GmbH) between 2011 and October 2018 and the digital droplet *KIT* D816V point mutation PCR assay (ddPCR) from Bio‐Rad Laboratories with a QX200 droplet reader (Bio‐Rad) according to the manufacturer's recommendations between November 2018 and 2020. Analyses were performed in a dedicated laboratory with expertise on CMD and PCR *KIT* point mutation analyses. Results were compared to the mRNA *KIT* D816V expression analysis in BM using a mismatch amplification real‐time PCR assay.^21^

### 2.5 Hereditary alpha tryptasemia

We assessed the frequency of HAT for patients with bsT between 7 and 11.4 ng/ml using genotyping of *TPSAB1* and *TPSB2* as described by Lyons et al.^22^ Patients with bsT <7 ng/ml were not assessed due to the rarity of HAT at such low bsT levels.^15^, ^22^, ^23^

### 2.6 Statistical methods

Patient characteristics were described using median, interquartile range or mean, standard deviation for continuous variables and number, percentage for categorical data. Statistical significance of differences between groups was determined using Mann‐Whitney U or independent *t*‐test for continuous and Chi‐square or Fisher's exact tests for categorical data.

Primarily we assessed performance of the REMA score in patients with normal and elevated bsT by calculating sensitivity and specificity with corresponding 95% confidence intervals (CI).

Secondarily, for patients with normal bsT univariate logistic regression analysis was performed to identify parameters associated with CMD. We used previously described methods for bsT, methylhistamine (MH) and methylimidazole acetic acid (MIMA) measurements.^19^ Data on continuous variables bsT, MH, MIMA, specific IgE against wasp venom (sIgE) and total IgE were non‐normally distributed and thus analysed after log transformation. BsT and MH were also analysed as categorical variables. The Youden's index was used to determine the optimal cut‐off for bsT, 6.3 ng/ml, and MH, 100 μmol/mol creatinine, on the ROC‐curve. *KIT* in PB was unsuitable for logistic regression analysis given the absence of false positives. Absence of multicollinearity was confirmed (variance inflation factor <5.0).

All parameters significantly associated with CMD (*p* < 0.05) or showing a trend towards significance (0.05 ≤ *p* < 0.10) were included in a forward Selection (Wald) multivariate logistic regression analysis. In addition, we tested patient characteristics gender and age. Dummy variables on (absence of) (reactions to) previous exposure to wasp venom were entered enp bloc.

Performance of *KIT* in PB was described by sensitivity and specificity with corresponding 95% CI. Outcomes of the HAT assay were given as frequencies.

The SPSS 23 statistical software package (SPSS) was used for statistical analyses, PRISM 8.4.2 (GraphPad Software) for graphical illustrations.

## 3 RESULTS

### 3.1 Study population

A total of 296 grade IV WVA patients visited our center within the inclusion period. The 186 included patients all underwent BME. Majority of included patients had a normal bsT (<11.4 ng/ml, *n* = 131). In total 35 of 131 patients with normal bsT (26.7%) were diagnosed with CMD, 24 with ISM (68.6%) and 11 with MMAS (31.4%). Among patients with elevated bsT 45 of 55 patients (81.8%) had CMD. Patients characteristics of CMD and non‐CMD patients are given in Table 1 for patients with normal bsT and Table S1 for patients with elevated bsT.

**TABLE 1:** Clinical characteristics of patients with normal basal serum tryptase (<11.4 ng/ml) with and without clonal mast cell disease

	Normal basal serum tryptase (*n* = 131)		
	Non‐CMD (*n* = 96)	CMD (*n* = 35)	*p* value
Male gender, *n* (%)	53 (55.2%)	22 (62.9%)	0.434
Age at index sting, years (IQR)	59.0 (47.0–64.0)	57.0 (46.0–64.0)	0.868
Age at CMD evaluation, years (IQR)	61.0 (51.0–66.0)	58.0 (52.0–66.0)	0.677
Diagnosis, *n* (%)	NA		NA
ISM		24 (68.6%)	
MMAS, CD25/CD2+, KIT+		4 (11.4%)	
MMAS, CD25/CD2+, KIT−		3 (8.8%)	
MMAS, CD25/CD2−, KIT+		4 (11.4%)	
Grade IVa SR upon index sting, *n* (%)	33 (34.4%)	4 (11.4%)	0.010*
Grade IVb SR upon index sting, *n* (%)	63 (65.6%)	31 (88.6%)	
Incontinence upon index sting, *n* (%)			0.012*
Absent	69 (76.7%)^a^	17 (53.1%)^a^	
Present	21 (23.3%)	15 (46.9%)	
Previously stung by wasps, *n*/*n* total (%)	80/95 (84.2%)	30/32 (93.8%)^a^	0.236
Previous SR to wasp venom, *n*/*n* total (%)	24/80 (30.0%)	16/30 (53.3%)	0.023*
Grade previous SR, *n* (%)			0.017*
I	4 (16.7%)	0 (0%)	
II	4 (16.7%)	0 (0%)	
III	1 (4.2%)	2 (12.5%)	
IVa	11 (45.8%)	5 (31.3%)	
IVb	4 (16.7%)	9 (56.3%)	
Grade previous SR, *n* (%)			0.009*
I–IVa	20 (83.3%)	7 (43.8%)	
IVb	4 (16.7%)	9 (56.3%)	
bsT, ng/mL (IQR)	4.6 (3.7–6.2)	6.7 (5.1–8.7)	<0.001*
MH, µmol/mol creatinine (IQR)	78.6 (60.9–97.7)	96.5 (73.4–115.9)^a^	0.025*
MIMA, mmol/mol creatinine (IQR)	1.6 (1.3–1.9)	1.6 (1.4–1.9)^a^	0.527
sIgE wasp, kU_A_/L (IQR)	6.8 (2.8–20.7)^a^	5.7 (0.9–22.0)^b^	0.304
Total IgE, kU_A_/L (IQR)	77 (41–178)^b^	57 (23–103)^c^	0.067
REMA score, *n* (%)			<0.001*
<2	74 (78.7%)	9 (28.1%)^a^	
≥2	20 (21.3%)	23 (71.9%)	
Peripheral *KIT*, *n* (%)			<0.001*
Positive	0 (0%)^d^	15 (55.6%)^d^	
Negative	41 (100%)	12 (44.4%)	

*Note*: Numbers given as number (%) or median (IQR). Bold values with * have *p* value <0.05.

Abbreviations: bsT, basal serum tryptase; CMD, clonal mast cell disease; ISM, indolent systemic mastocytosis; MH, methylhistamine; MIMA, methylimidazole acetic acid; MMAS, monoclonal mast cell activation syndrome; SR, systemic reaction.

^a^ 5%–10% missing data.

^b^ 10%–20% missing data.

^c^ 20%–50% missing data.

^d^ >50% missing data.

*p < 0.05.

**FIGURE 1:**
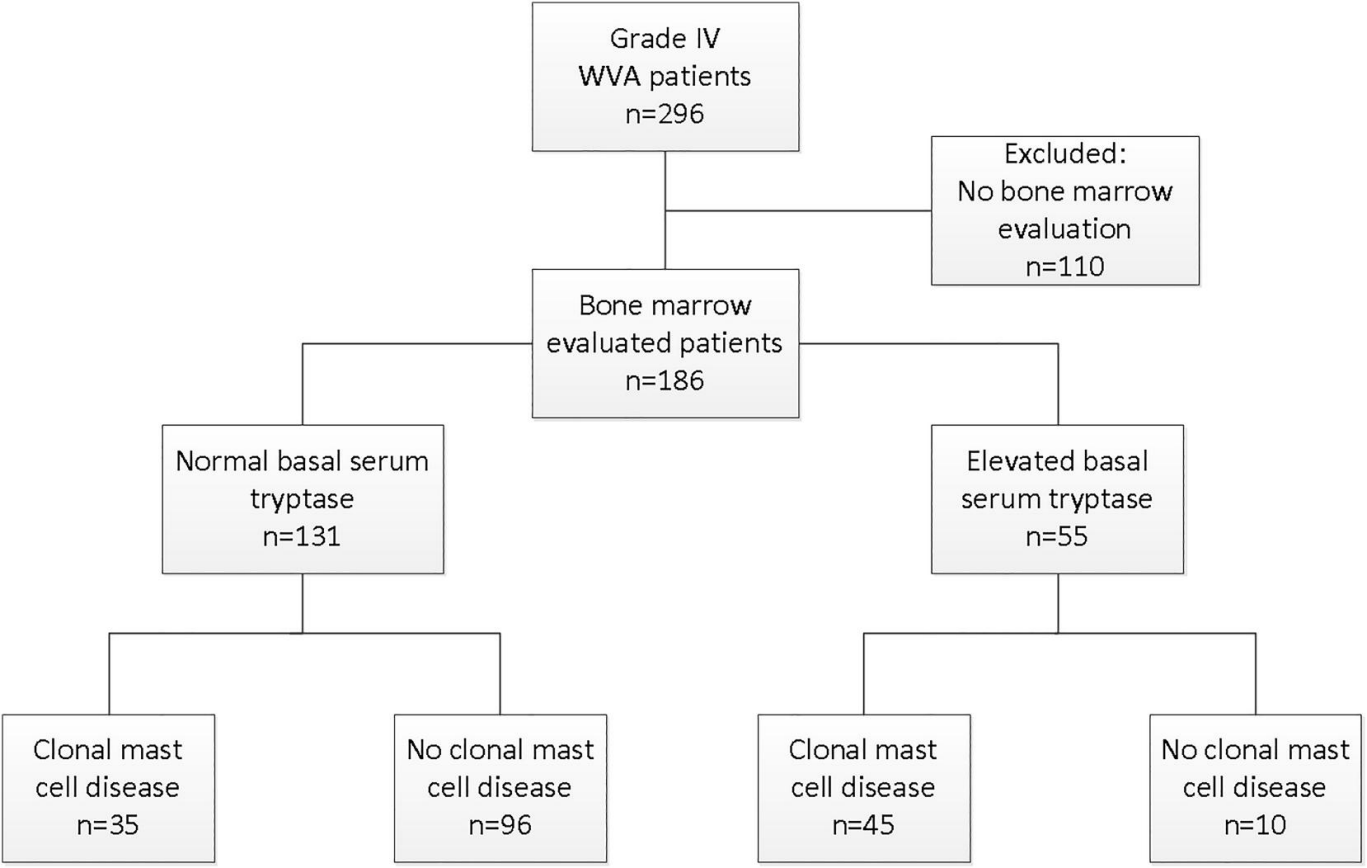
Flowchart of in‐ and exclusion, diagnostic results split up for normal (<11.4 ng/ml) and elevated (≥11.4 ng/ml) basal serum tryptase levels

Patients were excluded if they had not undergone BME (*n* = 110). These were mainly patients with normal bsT (*n* = 105) who visited between 2011 and 2016. A flowchart of in‐ and exclusion is shown in Figure 1. Patient characteristics of in‐ and excluded patients with normal bsT are shown in Table 2. The included cohort has an overrepresentation of grade IVb reactions. Physicians were apparently more hesitant in to perform BME in less severe cases.

**TABLE 2:** Clinical characteristics of patients with normal basal serum tryptase (<11.4 ng/ml) who were included in the study versus patients who were excluded based on the absence of bone marrow evaluation

	Included patient population (*n* = 131)	Excluded patient population, due to lack of BME (*n* = 105)	*p*‐value
Male gender, *n* (%)	75 (57.3%)	66 (62.9%)	0.383
Age at index sting, years (IQR)	58.0 (47.0–64.0)	57.0 (41.5–67.0)	0.728
Grade IVa SR upon index sting, *n* (%)	37 (28.2%)	78 (74.3%)	<0.001*
Grade IVb SR upon index sting, *n* (%)	94 (71.8%)	27 (25.7%)	
Incontinence upon index sting, *n* (%)			0.006*
Absent	86 (70.5%)^a^	82 (86.3%)^a^	
Present	36 (29.5%)	13 (13.7%)	
Previously stung by wasps, *n*/*n* total (%)	110/127 (86.6%)	82/94 (87.2%)^b^	0.893
Previous SR to wasp venom, *n*/*n* total (%)	40/110 (36.4%)	24/81 (29.6%)^e^	0.330
Grade previous SR, *n* (%)			0.458
I	4 (10.0%)	3 (13.0%)	
II	4 (10.0%)	5 (21.7%)	
III	3 (7.5%)	3 (13.0%)	
IVa	16 (40.0%)	5 (21.7%)	
IVb	13 (32.5%)	7 (30.4%)	
Grade previous SR, *n* (%)			
I–IVa	27 (67.5%)	16 (69.6%)	0.865
IVb	13 (32.5%)	7 (30.4%)	
bsT, ng/mL (IQR)	5.1 (4.0–6.7)	4.9 (3.9–6.7)	0.574
MH, µmol/mol creatinine (IQR)	83 (61–103)	90 (71–121)^b^	0.043*
MIMA, mmol/mol creatinine (IQR)	1.6 (1.3–1.9)	1.5 (1.2–1.9)^d^	0.674
sIgE wasp, kU_A_/L (IQR)	6.8 (2.2–20.9)^a^	5.1 (1.6–16.5)	0.259
Total IgE, kU_A_/L (IQR)	70 (35–159)^b^	68 (32–160)^a^	0.918
REMA score, *n* (%)			0.859
<2	83 (65.9%)	67 (67.0%)	
≥2	43 (34.1%)	33 (33.0%)	
Peripheral *KIT*, *n* (%)			0.034*
Positive	15 (22.1%)	0 (0%)	
Negative	53 (77.9%)^c^	19 (100%)^d^	

*Note*: Numbers given as number (%) or median (IQR). Bold values with * have *p* value <0.05.

Abbreviations: BME, bone marrow evaluation; bsT, basal serum tryptase; CMD, clonal mast cell disease; ISM, indolent systemic mastocytosis; MH, methylhistamine; MIMA, methylimidazole acetic acid; MMAS, monoclonal mast cell activation syndrome; SR, systemic reaction.

^a^ 5%–10% missing data.

^b^ 10%–20% missing data.

^c^ 20%–50% missing data.

^d^ 50% missing data.

^e^ In one patient the reaction upon previous exposure was unknown.

### 3.2 REMA score

**FIGURE 2:**
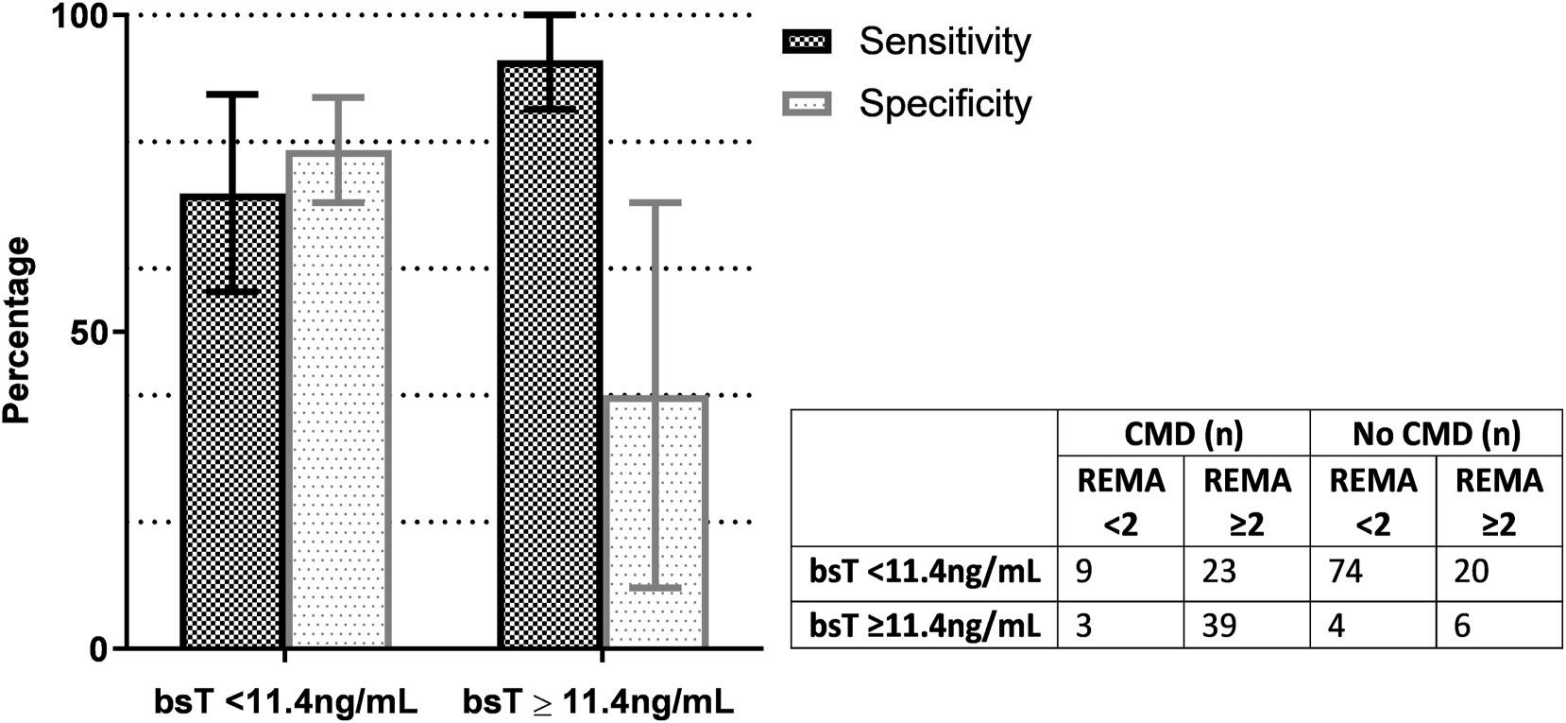
Sensitivity and specificity of the REMA score with 95% confidence intervals split up for normal (<11.4 ng/ml) and elevated (≥11.4 ng/ml) basal serum tryptase

The REMA score was available for 126/131 patients with normal and 52/55 patients with elevated bsT. The 8 patients with missing REMA scores lacked data on skin symptoms during the SR. Figure 2 shows sensitivity and specificity of the REMA score per tryptase category.

Sensitivity was higher in patients with elevated bsT levels, 92.3% (95% CI 85.1%–100%). Specificity was relatively low, 40.0% (95% CI 9.6%–70.4%). The wide CI resulted from the high prevalence of CMD, 81.8%, and the consequently low number of non‐CMD patients.

In patients with normal bsT sensitivity of the REMA score was 71.9% (95% CI 56.0%–87.8%), specificity was 78.7% (95% CI 70.3%–87.2%). The REMA score can thus identify a reasonably large share of patients at risk of CMD within the normal bsT subpopulation. Nevertheless, an important proportion of CMD patients (9/32, 28.1%) would be missed if relying solely on the REMA score. Therefore, we assessed if there were additional parameters independently associated with CMD that could further improve sensitivity in the subpopulation with normal bsT.

### 3.3 Other independently associated parameters

This is the first study that performs a multivariate analysis of parameters independently associated with CMD in a BM evaluated cohort of patients with normal bsT. Univariate analysis, see Table 3, showed that the following parameters were significantly associated with CMD: loss of consciousness (grade IVb) at the index sting (*p* = 0.014) or upon previous exposure (*p* = 0.001), absent skin features during the SR (in this population a reflection of the REMA score) (*p* < 0.001), incontinence during the SR (*p* = 0.0014), bsT levels (continuous and categorical [*p* < 0.001]) and MH (categorical [*p* = 0.004]). Total IgE levels showed a trend towards significance (*p* = 0.056).

**TABLE 3:** Uni‐ and multivariate logistic regression analysis in patients with normal basal serum tryptase (<11.4 ng/ml)

	Univariate		Multivariate	
	OR (95% CI)	*p* value	OR (95% CI)	*p* value
Male gender	1.373 (0.620–3.043)	0.434	3.207 (0.988–10.409)	0.052
Age at index sting	1.001 (0.973–1.029)	0.959		
Age at evaluation for CMD	0.993 (0.963–1.025)	0.678		
Grade IVb SR upon index sting	4.060 (1.320–12.482)	0.014*	8.345 (1.640–42.474)	0.011*
REMA score ≥2/Absent skin features during SR upon index sting	9.456 (3.786–23.616)	<0.001*	6.834 (2.152–21.695)	0.001*
Incontinence upon index sting^a^	2.899 (1.240–6.776)	0.014*		
No previous reaction (reference)				
OR no previous exposure	0.533 (0.109–2.609)	0.438	0.281 (0.027–2.908)	0.287
OR previous SR grade I–IVa	1.400 (0.494–3.965)	0.526	1.422 (0.371–5.603)	0.597
OR previous SR grade IVb	9.000 (2.415–33.535)	0.001*	16.092 (2.362–109.643)	0.005*
Log10 bsT	693.706 (30.746–15,651.545)	<0.001*		
bsT, ≥6.3 ng/ml	5.700 (2.469–13.159)	<0.001*	4.155 (1.344–12.845)	0.013*
Log10 MH	8.736 (0.684–111.635)	0.095		
MH, >100 μmol/mol creatinine	3.482 (1.499–8.088)	0.004*		
Log10 MIMA	1.314 (0.055–31.133)	0.866		
Log10 sIgE wasp^a^	0.687 (0.370–1.274)	0.234		
Log10 total IgE^b^	0.389 (0.148–1.023)	0.056		

*Note*: Data on 126 of 131 patients were available for the multivariate analysis. Nagelkerke *R* ^2^ of this analysis is 0.503. Bold values with * have *p* value <0.05.

Abbreviations: bsT, basal serum tryptase; CMD, clonal mast cell disease; MH, methylhistamine; MIMA, methylimidazole acetic acid; OR, odds ratio; SR, systemic reaction.

^a^ 5%–10% missing data.

^b^ 10%–20% missing data.

Subsequent multivariate regression analysis, see Table 3, identified absent skin features (*p* = 0.001), grade IVb reactions at the index sting (= 0.011), bsT ≥6.3 ng/ml (*p* = 0.013), and grade IVb reactions upon previous exposure (*p* = 0.005) as parameters that were independently associated with CMD. Male gender showed a trend towards significance (*p* = 0.052). Nagelkerke *R* ^2^ of this multivariate model was 0.503, that is, these parameters explained 50.3% of the variation in CMD.

A major finding of this analysis is that the REMA score, also in patients with a normal tryptase, explains the largest proportion of variation of this model, Nagelkerke *R* ^2^ 0.266. This emphasizes that also in patients with normal bsT absent skin features during the systemic reaction are a main predictor of CMD. Furthermore, the additional identified parameters all appear to be in line with parameters reflected in the REMA score: also within the normal bsT population more severe reactions and higher bsT levels reflect an increased risk of CMD. We were not able to convert these findings into an adaptation of the REMA score for this population due to lack of power.

### 3.4 *KIT* D816V mutation analysis in peripheral blood

**FIGURE 3:**
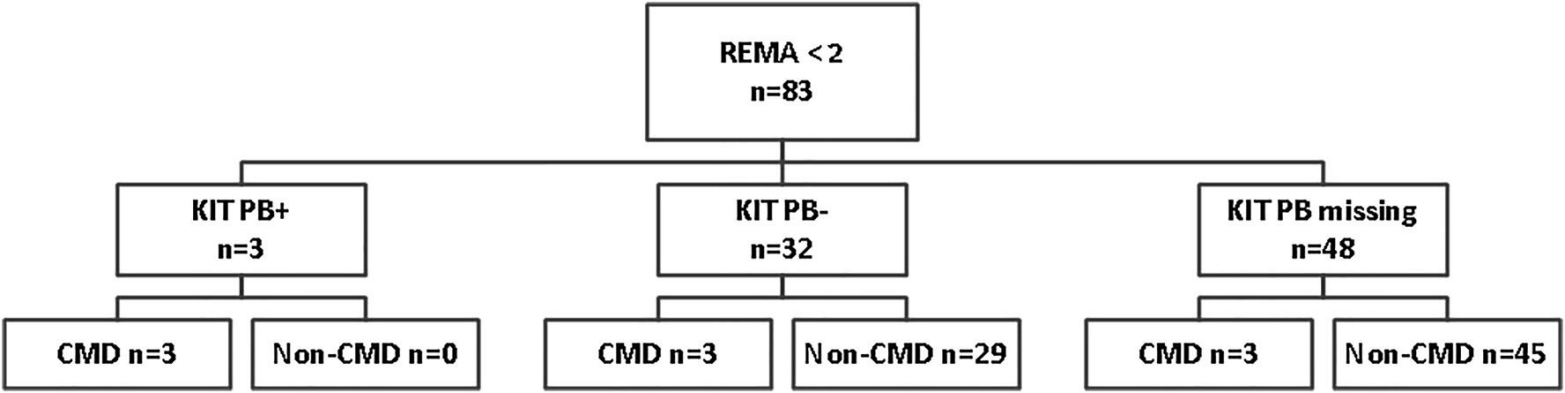
Results *KIT* D816V analysis in peripheral blood in patients with normal basal serum tryptase and a low REMA score (<2)

*KIT* D816V mutation analysis in PB was available for 68 WVA patients with normal bsT, 27 patients with CMD and 41 non‐CMD patients. Sensitivity of this analysis was 55.6% (95% CI 36.4%–74.7%) and was similar for both types of assays used. Specificity of *KIT* in PB was 100%. Due to the low sensitivity *KIT* in PB is not suitable as alternative for the REMA score. However, due to the high specificity it could be of added value as detection method in patients classified as low risk by the REMA score. As shown in Figure 3, *KIT* in PB detected 3 of 6 CMD patients that had a REMA score <2.

A remarkable observation was that sensitivity of the *KIT* analysis in PB was particularly low in patients diagnosed with MMAS rather than ISM. Only 2 of the 10 (20%) MMAS patients tested positive on *KIT* analysis in PB, against 13 of 17 (76.5%) ISM patients. Five of the eight missed MMAS patients tested positive for the *KIT* D816V mutation in BM.

### 3.5 Hereditary alpha tryptasemia

Lastly we assessed the prevalence of hereditary alpha tryptasemia in a patients with a bsT between 7 and 11.4 ng/ml. Results of the assay were available for 28 of the 29 patients in this range. Sixteen of 28 were diagnosed with CMD, 12 ISM and 4 MMAS. A duplication of TPSAB1 was found in 3 of 28 patients (10.7%), 2 in CMD patients (12.5%, both diagnosed with ISM), 1 in a non‐CMD patient (8.3%). Although numbers are too low for definitive conclusions this assay seemingly has no (added) value for CMD risk assessment within this population.

## 4 DISCUSSION

This is the first study that systematically assessed detection methods for CMD in a large group of BM evaluated patients with severe WVA and normal bsT levels. We found that also in normal bsT patients the REMA score is important and reliable in detection of CMD. Sensitivity of this score is 72%, specificity 79%. Use of the REMA score in this population can therefore greatly reduce the number of required BMEs. In addition, we showed that the number of missed CMD cases could be further decreased if patients with low REMA scores would undergo *KIT* D816V mutation analysis in peripheral blood. Screening for HAT did not appear to be of added value for CMD risk assessment. We wish to emphasize that REMA score or any of the other tools are no replacement for BME as not all cases are detected.

Although sensitivity of the REMA score was high in this population with normal bsT, it was clearly lower than in patients with elevated bsT. Sensitivity in patients with elevated bsT levels in the current Dutch cohort was consistent with the findings in the Spanish cohort: 92% in both groups.^10^ The diminished reliability of the REMA score thus specifically applied to the patient group with normal bsT. This lower sensitivity is not surprising as REMA score deducts 1 point for bsT levels <15 ng/mL. As a result, detection of CMD relies solely on presence or absence of skin features during the SR in this subgroup. Moreover, information on this parameter is not always available and might be prone to recall bias.

Therefore, the results from the multivariate analysis are highly interesting to see if other clinical parameters could optimize detection of CMD in the normal bsT population. We identified loss of consciousness during the SR and higher bsT levels (≥6.3 ng/ml) as parameters that were also independently associated with CMD. These findings underline the relevance of the parameters of the REMA score in detecting CMD. Both the REMA score, as well as loss of consciousness reflect an increased risk of CMD in patients with more severe anaphylactic reactions.

Our study has two limitations. Firstly, we were not able to convert the findings of the multivariate analysis into an adaptation of the REMA score due to lack of power. Secondly, there was a relative overrepresentation of grade IVb reactions as compared to the patients who were excluded because they did not undergo BME. This may hamper the generalizability of our results to the full grade IV cohort. However, this is the first large, BM controlled cohort to assess risk factors in multivariate analysis. In spite of the selection bias, the results are in line with a previous, non‐BME controlled study that compared patient characteristics of grade IV patients with normal bsT with positive and negative *KIT* mutation analysis in PB.^14^ In that study unconsciousness and higher tryptase levels were associated with a positive *KIT* in PB, and thus indirectly with CMD.^14^ The consistency of our findings with literature makes these results highly interesting for future prospective studies in normal bsT cohorts.

Our findings on the *KIT* assay in PB confirmed findings of previous studies regarding the high specificity (100%).^11^, ^12^ Therefore it could have added value as additional screening method in patients with a low REMA score. Here and in previous reports several patients with low REMA scores who tested positive for *KIT* in PB are described.^12^, ^13^ Whether routine use of *KIT* in PB would be a cost‐effective and whether the remaining risk of missing diagnoses of CMD is acceptable should be subject of international discussion.

The differences in sensitivity between the Danish study on one hand, and our data and the Belgian data on the other hand is striking. The use of another *KIT* assay for part of the analyses; that is, a ddPCR instead of qPCR, could not explain the lower *KIT* sensitivity (data not shown), in line with Greiner et al.^24^ who also found highly concordant performances for both assays. The difference might be explained by the inclusion of only one MMAS patient in the Danish cohort,^11^ while sensitivity in the current and Belgian cohort was particularly low in MMAS patients.^12^ The reduced sensitivity in MMAS patients could reflect a lower mast cell burden and/or lower multilineage *KIT* positivity, which would be in accordance with the less pronounced aberrations found in their BM. However, current consensus is that MMAS patients should be treated in the same manner as ISM patients, making diagnosis equally important.

Given the high positive predictive value of the *KIT* in PB, one could postulate that a positive *KIT* in PB could replace BME. According to current guidelines BME is indicated in all patients suspected of CMD.^18^ As a positive *KIT* mutation in PB appears to affirm CMD, it could potentially be sufficient basis for lifelong VIT. The BME would than primarily serve to detect underlying associated haematological disease. The prevalence of associated haematological disease within CMD WVA patients has not yet been systematically evaluated.

Lastly, this study evaluated the frequency of HAT and its potential added value in detecting CMD in this subpopulation. Although numbers were too low to draw conclusions, frequency in the normal bsT range is low^15^, ^22^ and HAT was found in both CMD and non‐CMD patients. Therefore, it seems it unlikely that this assay will be of added value in detecting CMD in this particular patient population.

In conclusion, we have shown that a combination of the REMA score and *KIT* in PB analysis is a reliable method for detection of CMD in severe wasp venom allergic patients with normal bsT. In addition we have shown that also within this subpopulation higher bsT levels and more severe SRs reflect an increased risk of CMD.

## AUTHOR CONTRIBUTIONS

**Merel C. Onnes**: Conceptualization (supporting); Data curation (lead); Formal analysis (lead); Investigation (lead); Methodology (equal); Project administration (lead); Visualization (lead); Writing – original draft (lead); Writing – review & editing (lead). **Abdulrazzaq Alheraky**: Formal analysis (equal). **Martijn C. Nawijn**: Conceptualization (supporting); Methodology (supporting); Supervision (supporting); Writing – review & editing (supporting). **Tim E. Sluijter**: Investigation (supporting). **Andre B. Mulder**: Formal analysis (supporting); Methodology (supporting); Writing – original draft (supporting); Writing – review & editing (supporting). **Suzanne Arends**: Methodology (equal); Supervision (supporting); Visualization (supporting); Writing – original draft (supporting); Writing – review & editing (supporting). **Hanneke N. G. Oude Elberink**: Conceptualization (lead); Data curation (equal); Methodology (equal); Supervision (lead); Writing – original draft (supporting); Writing – review & editing (supporting).

## CONFLICT OF INTEREST

Hanneke N. G. Oude Elberink has received fees for delivering lectures from ALK‐Abelló, Mylan, Sanofi Genzyme and Novartis; has received consultancy fees from ALK‐Abello, Novartis, Blueprint and Sanofi Genzyme; has received research support from Novartis, Mylan, ALK‐Abello, Aimmune, Takeda and Blueprint; and has received payment for developing educational presentations from ALK‐Abello and Mylan. The other authors declare that they have no conflicts of interest.

## Supporting information

Table S1
