# Supplementary material for: Detection of clonal mast cell disease in wasp venom allergic patients with normal tryptase
Source: Clin Transl Allergy. 2022 Sep 7;12(9):e12174. doi: 10.1002/clt2.12174 (PMC9449818; doi:10.1002/clt2.12174)
Supplement: Supplementary file 1 — Table S1 [file CLT2-12-e12174-s001.docx]

**Supplementary Table 1** *Clinical characteristics of patients with elevated basal serum tryptase (≥11.4ng/mL) with and without clonal mast cell disease. Numbers given as number (%) or median (IQR). *P<0.05. bsT = basal serum tryptase CMD = clonal mast cell disease, ISM = indolent systemic mastocytosis, MH = methylhistamine, MIMA = methylimidazole acetic acid, MMAS = monoclonal mast cell activation syndrome, SR = systemic reaction.* ^†^*5-10% missing data,* ^‡^*10-20% missing data,* ^§^*20-50% missing data,* ^¶^*>50% missing data.*

|  | **Elevated basal serum tryptase (n=55)** | | |
| --- | --- | --- | --- |
|  | **Non-CMD**  **(n=10)** | **CMD**  **(n=45)** | **P-value** |
| **Male gender, n (%)** | 7 (70.0%) | 34 (75.6%) | 0.703 |
| **Age at index sting, years (IQR)** | 55.5 (47.0 - 67.3) | 57.0 (47.5 – 65.0) | 0.844 |
| **Age at CMD evaluation, years (IQR)** | 59.5 (47.8 – 68.8) | 58.0 (49.5 – 66.0) | 0.615 |
| **Diagnosis, n (%)**  ***ISM***  ***MMAS, CD25/CD2+, KIT+***  ***MMAS, CD25/CD2+, KIT-***  ***MMAS, CD25/CD2-, KIT+*** | NA | 41 (91.1%)  1 (2.2%)  1 (2.2%)  2 (4.4%) | NA |
| **Grade IVa SR upon index sting, n (%)**  **Grade IVb SR upon index sting, n (%)** | 2 (20.0%)  8 (80.0%) | 6 (13.3%)  39 (86.7%) | 0.627 |
| **Incontinence upon index sting, n (%)**  ***Absent***  ***Present*** | 8 (100.0%)^§^  0 (0.0%) | 15 (45.5%)^§^  18 (54.5%) | **0.006*** |
| **Previously stung by wasps, n/n total (%)** | 8/9 (88.9%)^‡^ | 37/45 (82.2%) | >0.999 |
| **Previous SR to wasp venom, n/n total (%)** | 1/8 (12.5%) | 19/37 (51.4%) | 0.059 |
| **Grade previous SR, n (%)**  ***I***  ***II***  ***III***  ***IVa***  ***IVb*** | 0 (0%)  0 (0%)  0 (0%)  0 (0%)  1 (100%) | 0 (0%)  3 (15.8%)  2 (10.5%)  3 (15.8%)  11 (57.9%) | >0.999 |
| **Grade previous SR, n (%)**  ***I-IVa***  ***IVb*** | 0 (0%)  1 (100%) | 8 (42.1%)  11 (57.9%) | >0.999 |
| **bsT, ng/mL (IQR)** | 15.8 (13.2 – 19.2) | 19.1 (14.5 – 27.8) | 0.124 |
| **MH, µmol/mol creatinine (IQR)** | 85 (60 – 96) | 136 (97 – 183) | **0.001*** |
| **MIMA, mmol/mol creatinine (IQR)** | 1.5 (1.2 – 3.0)^‡^ | 2.3 (1.9 – 2.9) | 0.127 |
| **sIgE wasp, kU_A_/L (IQR)** | 12.4 (5.6 – 51.8)^‡^ | 1.3 (0.7 – 5.2)^§^ | **0.001*** |
| **Total IgE, kU_A_/L (IQR)** | 77 (19 – 148) ^‡^ | 22 (11 – 121)^§^ | 0.250 |
| **REMA score, n (%)**  ***<2***  ***≥2*** | 4 (40%)  6 (60%) | 3 (7.1%)^‡^  39 (92.9%) | **0.020*** |
| **Peripheral *KIT* , n (%)**  ***Positive***  ***Negative*** | 0 (0%)^¶^  3 (100%) | 18 (75.0%)^§^  6 (25.0%) | **0.029*** |
